# An Updated Meta-Analysis on the Association of MDM2 SNP309 Polymorphism with Colorectal Cancer Risk

**DOI:** 10.1371/journal.pone.0076031

**Published:** 2013-09-30

**Authors:** Xue Qin, Qiliu Peng, Weizhong Tang, Xianjun Lao, Zhiping Chen, Hao Lai, Yan Deng, Cuiju Mo, Jingzhe Sui, Junrong Wu, Limin Zhai, Shi Yang, Shan Li, Jinmin Zhao

**Affiliations:** 1 Department of Clinical Laboratory, First Affiliated Hospital of Guangxi Medical University, Nanning, Guangxi, China; 2 Department of Anal and Colorectal Surgery, First Affiliated Hospital of Guangxi Medical University, Nanning, Guangxi, China; 3 Department of Occupational Health and Environmental Health, School of Public Health at Guangxi Medical University, Nanning, Guangxi, China; 4 Department of Gastrointestinal Surgery, Tumor Hospital of Guangxi Medical University, Nanning, Guangxi, China; 5 Department of Orthopedic Trauma Surgery, First Affiliated Hospital of Guangxi Medical University, Nanning, Guangxi, China; Sanjay Gandhi Medical Institute, India

## Abstract

**Background:**

The mouse double minute 2 (MDM2) gene encodes a phosphoprotein that interacts with P53 and negatively regulates its activity. The SNP309 polymorphism (T-G) in the promoter of MDM2 gene has been reported to be associated with enhanced MDM2 expression and tumor development. Studies investigating the association between MDM2 SNP309 polymorphism and colorectal cancer (CRC) risk reported conflicting results. We performed a meta-analysis of all available studies to explore the association of this polymorphism with CRC risk.

**Methods:**

All studies published up to July 2013 on the association between MDM2 SNP309 polymorphism and CRC risk were identified by searching electronic databases PubMed, EMBASE, and Chinese Biomedical Literature database (CBM) databases. The association between the MDM2 SNP309 polymorphism and CRC risk was assessed by odds ratios (ORs) together with their 95% confidence intervals (CIs).

**Results:**

A total of 14 case-control studies including 4460 CRC cases and 4828 controls were identified. We did not find a significant association between the MDM2 SNP309 polymorphism and CRC risk in all genetic models in overall population. However, in subgroup analysis by ethnicity, significant associations were found in Asians (TG vs. TT: OR = 1.197, 95% CI = 1.055–1.358, P=0.005; GG+TG vs. TT: OR = 1.246, 95% CI = 1.106–1.404, P=0.000) and Africans. When stratified by HWE in controls, significantly increased risk was also found among the studies consistent with HWE (TG vs. TT: OR = 1.166, 95% CI = 1.037–1.311, P= 0.010). In subgroup analysis according to p53 mutation status, and gender, no any significant association was detected.

**Conclusions:**

The present meta-analysis suggests that the MDM2 is a candidate gene for CRC susceptibility. The MDM2 SNP309 polymorphism may be a risk factor for CRC in Asians.

## Introduction

Colorectal cancer (CRC) is one of the most common forms of cancer and is the third leading cause of cancer-related death worldwide [1]. In Europe and the USA, CRC represents one of the primary causes of cancer deaths [1,2]. In Asia, CRC is the fourth leading cause of mortality by cancer, and its incidence is increasing [3]. In recent years, the incidence of CRC is increasing in China, which accounts for about 6.5% of total cancers in urban areas and 4.6% in rural areas [4]. Previous epidemiological studies have identified dietary factors, such as consumption of meat, especially red meat, and cigarette smoking as possible risk factors for the development of CRC [5,6]. However, most individuals with these known dietary risk factors never develop CRC while many CRC cases develop among individuals without those known risk factors. The exact mechanism of CRC carcinogenesis is still far from clear.

The murine double minute-2 (MDM2), a key negative regulator of the P53 tumor suppressor pathway, has been suggested to be implicated in a variety of cancers [7]. Evidence indicated that MDM2 can bind directly to P53 protein and inhibit its activity, thus resulting in its degradation via the ubiquitination pathway [8]. A single nucleotide polymorphism (SNP) in the promoter region of MDM2, SNP T309G (rs2279744), has been identified and was demonstrated to up-regulate the expression of MDM2 via a greater affinity for the SP1 transcription factor. Consequently, individuals carrying the GG genotype of the MDM2 SNP309 polymorphism were found to have higher MDM2 levels, which led to attenuation of the TP53 pathway and acceleration of tumor formation in humans [9]. It was reported that the increase in MDM2 results in direct inhibition of p53 transcriptional activity, enabling damaged cells to escape the cell-cycle checkpoint and become carcinogenic [10]. Hence, it is biologically reasonable to hypothesize a potential relationship between the MDM2 SNP309 polymorphism and CRC risk.

Over the last two decades, a number of molecular epidemiological studies have been conducted to investigate the association between the MDM2 SNP309 polymorphism and CRC risk, but the results remain inconsistent. In addition, previous two meta-analyses on this issue also generated conflicting results [11,12]. Small genetic association studies have various designs, different methodology, and insufficient power, and could inevitably increase the risk that chance could be responsible for their conclusions, while combining data from all eligible studies by meta-analysis has the advantage of reducing random error and obtaining precise estimates for some potential genetic associations. Therefore, in this study, we conducted a quantitative updated meta-analysis including all eligible data up to July 2013, increasing statistical power to derive a more precise estimation of the relationship.

## Materials and Methods

### Search strategy

This study was performed according to the proposal of Meta-analysis of Observational Studies in Epidemiology group (MOOSE) [13]. We conducted a comprehensive literature search in PubMed, Embase, and Chinese Biomedical Literature database (CBM) databases up to July 01, 2013 using the following search strategy: (“colorectal cancer”, “CRC”, “colon cancer” or “rectum cancer”) and (“Murine double minute 2”, or “MDM2”) and (“polymorphism”, “variation”, “mutation”, “genotype”, or “genetic polymorphism”). There was no restriction on time period, sample size, population, language, or type of report. All eligible studies were retrieved and their references were checked for other relevant studies. The literature retrieval was performed in duplication by two independent reviewers (Xue Qin and Qiliu Peng). When multiple publications reported on the same or overlapping data, we chose the most recent or largest population. When a study reported the results on different subpopulations, we treated it as separate studies in the meta-analysis.

### Selection criteria

Studies included in the meta-analysis were required to meet the following criteria: (1) Case–control studies which evaluated the association between MDM2 SNP309 polymorphism and CRC risk; (2) used an unrelated case–control design; (3) had an odds ratio (OR) with 95% confidence interval (CI) or other available data for estimating OR (95% CI); and (4) control population did not contain malignant tumor patients. Conference abstracts, case reports, editorials, review articles, and letters were excluded.

### Data extraction

Two reviewers (Xue Qin and Qiliu Peng) independently reviewed and extracted data from all eligible studies. Data extracted from eligible studies included the first author, year of publication, country of origin, ethnicity, genotyping method, matching criteria, source of control, CRC diagnosis criteria, total numbers of cases and controls and genotype frequencies of cases and controls. Ethnic backgrounds were categorized as Caucasian, Asian, and African. When a study did not state the ethnic descendent or if it was not possible to separate participants according to such phenotype, the group reported was termed as “mixed ethnicity”. To ensure the accuracy of the extracted information, the two investigators checked the data extraction results and reached consensus on all of the data extracted. If different results were generated, they would check the data again and have a discussion to come to an agreement. A third reviewer (Li Shan) was invited to the discussion if disagreement still existed.

### Methodological quality assessment

Methodological quality was independently assessed by two reviewers (Xue Qin and Qiliu Peng), according to a set of predefined criteria (Table 1) based on the scale of Thakkinstian et al. [14]. The revised criteria cover the credibility of controls, the representativeness of cases, assessment of CRC, genotyping examination, Hardy-Weinberg equilibrium in the control population, and association assessment. Disagreements were resolved by consensus. Scores ranged from 0 (lowest) to 12 (highest). Articles with scores less than 8 were considered ‘‘low -quality’’ studies, whereas those with scores equal to or higher than 8 were considered “high-quality’’ studies.

**Table 1:** Scale for Quality Assessment.

Criteria	Score
Representativeness of cases	
Selected from population or cancer registry	2
Selected from any gastroenterology /surgery service	1
Selected without clearly defined sampling frame or with extensive inclusion/exclusion criteria	0
Credibility of controls	
Population- or neighbor- based	3
Blood donors or volunteers	2
Hospital-based (cancer-free patients)	1
Healthy volunteers, but without total description	0.5
Gastroenterology patients	0.25
Not described	0
Ascertainment of colorectal cancer	
Histological or pathological confirmation	2
Diagnosis of colorectal cancer by patient medical record	1
Not described	0
Genotyping examination	
Genotyping done under ‘‘blinded’’ condition	1
Unblinded or not mentioned	0
Hardy-Weinberg equilibrium	
Hardy-Weinberg equilibrium in controls	2
Hardy-Weinberg disequilibrium in controls	1
No checking for Hardy-Weinberg disequilibrium	0
Association assessment	
Assess association between genotypes and colorectal cancer with appropriate statistics and adjustment for confounders	2
Assess association between genotypes and colorectal cancer with appropriate statistics without adjustment for confounders	1
Inappropriate statistics used	0

### Statistical analysis

The strength of the association between MDM2 SNP309 polymorphism and CRC risk was measured by odds ratios (ORs) with 95% confidence intervals (CIs). The significance of the pooled OR was determined by Z test and a *p* value of less than 0.05 was considered significant. We assessed the association of MDM2 SNP309 polymorphism with CRC risk using additive models (GG vs. TT and TG vs. TT), recessive model (GG vs. TG+TT), and dominant model (GG+TG vs. TT).

The *Q* test and *I* ^2^ statistics were used to assess the statistical heterogeneity among studies [15,16]. If the result of the *Q* test was *P*_*Q*_ < 0.1, indicating the presence of heterogeneity, a random-effects model (the DerSimonian and Laird method) was used to estimate the summary ORs [17]; otherwise, when the result of the *Q* test was *P*_*Q*_ ≥ 0.1, indicating the absence of heterogeneity, the ﬁxed-effects model (the Mantel–Haenszel method) was used [18]. To explore the sources of heterogeneity among studies, we performed logistic metaregression and subgroup analyses. The following study characteristics were included as covariates in the metaregression analysis: genotyping methods (PCR-RFLP versus not PCR-RFLP), ethnicity (Caucasian population versus Asian population), source of controls (Hospital-based versus Population-based), Quality scores (High-quality versus Low-quality) and CRC diagnosis (pathologically or histologically confirmed versus other diagnosis criteria). Subgroup analyses were conducted by ethnicity, p53 mutation status, gender, and HWE in controls. Galbraith plots analysis was performed for further exploration of the heterogeneity.

Sensitivity analysis was performed by sequential omission of individual studies. Publication bias was evaluated using a funnel plot and Egger’s regression asymmetry test [19]. If publication bias existed, the Duval and Tweedie non-parametric “trim and ﬁll” method was used to adjust for it [20]. The distribution of the genotypes in the control population was tested for HWE using a goodness-of-fit Chi-square test. All analyses were performed using Stata software, version 12.0 (Stata Corp., College Station, TX). All *p* values were two-sided. To ensure the reliability and the accuracy of the results, two authors entered the data into the statistical software programs independently with the same results.

## Results

### Characteristics of studies

**Figure 1:**
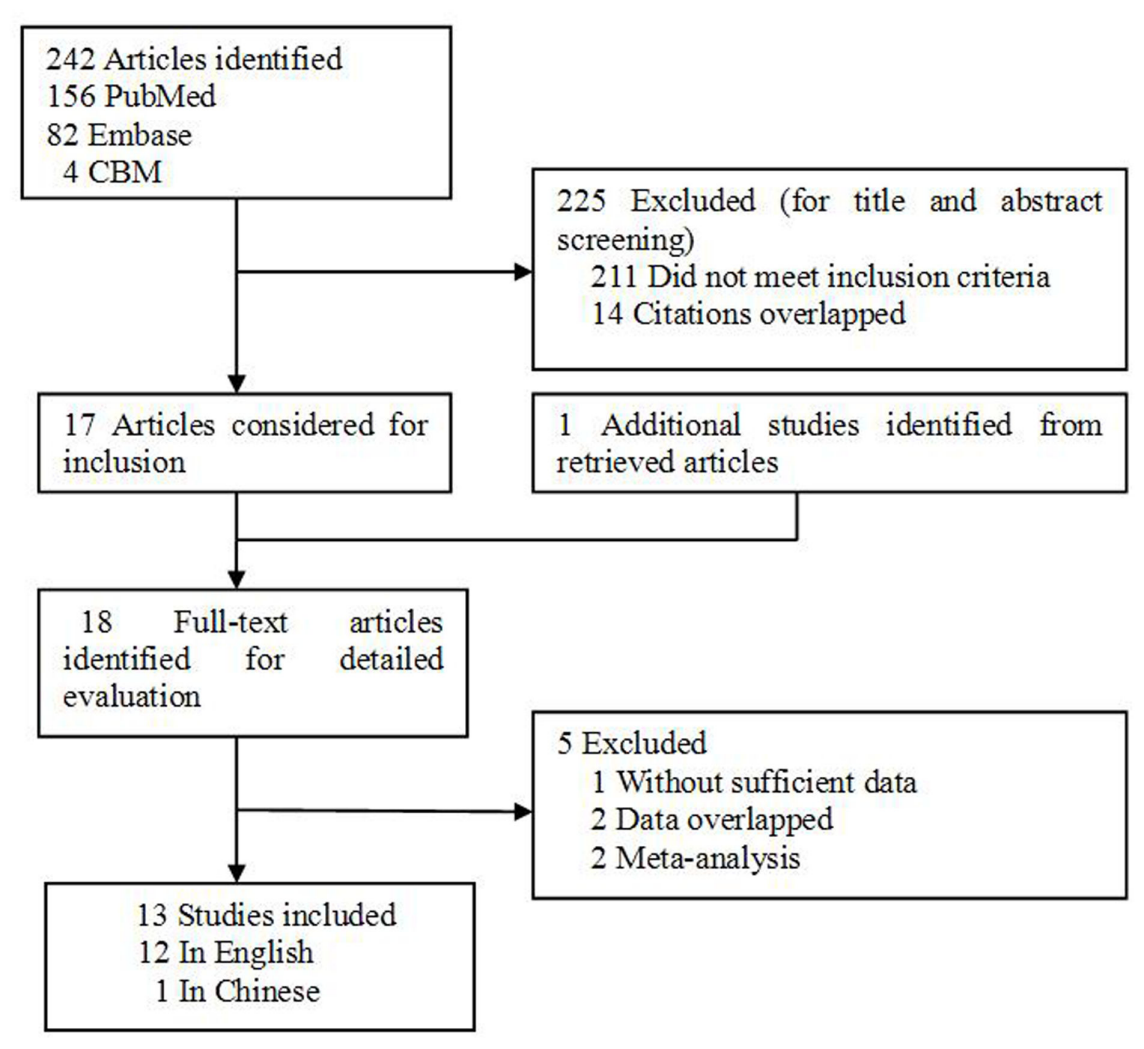
Flowchart of selection of studies for inclusion in meta-analysis.

Based on our search criteria, 242 individual records were found, but only 17 full-text publications were preliminarily identified for further detailed evaluation (Figure 1). According to the exclusion criteria, 5 publications were excluded including 2 publications containing overlapping data [21,22], 1 for not presenting sufficient data for calculating OR and 95% CI [23], and 2 were meta-analysis [11,12]. Manual search of references cited in the eligible studies identified 1 additional article [24]. As a result, a total of 13 relevant studies including 12 English articles [24,25,26,27,28,29,30,31,32,33,34,35], and 1 Chinese study [36] met the inclusion criteria for the meta-analysis. Among them, one of the eligible studies contained data on two different populations (Finnish and American) [26] and we treated it independently. Therefore, a total of 14 separate comparisons including a total of 4460 CRC cases and 4828 controls were finally included in our meta-analysis. The main characteristics of the studies were presented in Table 2. Of all the eligible studies, 7 were conducted in Caucasian populations, 6 were in Asians, and 1 was in Africans. Six studies were population–based and 8 were hospital–based studies. All studies used validated methods including PCR-RFLP, PCR-SSCP, TaqMan assay, FISH, MALDI-TOF, and On-Chip Electrophoresis to genotype the MDM2 SNP309 polymorphism. The CRC cases were histologically confirmed or pathologically confirmed in 9 of the eligible studies. The genotype distributions of the controls in 5 studies [26,28,32,34,36] were not consistent with HWE for MDM2 SNP309 polymorphism.

**Table 2:** Characteristics of studies included in this meta-analysis.

First author (Year)	Country	Ethnicity	Sample size (case/control)	Genotyping methods	Matching criteria	Source of control	CRC diagnosis	Quality scores	HWE(*P* value)
Alhopuro 2005	Finland	Caucasian	969/185	PCR-RFLP	Region	PB	HC	8	0.282
Sotamaa1 2005	Finland	Caucasian	121/209	PCR-RFLP	Region, gender	PB	NA	8	0.351
Sotamaa2 2005	America	Caucasian	30/138	PCR-RFLP	Region, gender	PB	NA	8	**0.004**
Menin 2006	Italy	Caucasian	153/92	PCR-SSCP	Region	PB	HC	5	0.689
Talseth 2006	Australia	Caucasian	116/98	TaqMan, Assay	NA	HB	NA	5	0.085
Alazzouzi 2007	Spain	Caucasian	152/184	PCR-SSCP	Ethnicity	HB	NA	4	**0.011**
Liu 2008	China	Asian	1000/1300	ARMS-PCR	Age, gender	HB	PC	10	0.757
Jin 2008	China	Asian	202/836	PCR-RFLP	Smoking, drinking,gender	PB	PC	9	**0.000**
Chen 2009	China	Asian	123/138	PCR-SSCP	NA	HB	NA	4	**0.017**
Sugano 2010	Japan	Asian	211/59	FISH	NA	HB	PC	5	0.604
Joshi 2011	Japan	Asian	685/778	PCR-RFLP	Age, gender	PB	HC	11	0.775
Zhang 2012	China	Asian	444/569	MALDI-TOF	Age, gender	HB	HC	8	0.928
Chaar 2012	Tunisia	African	167/167	On-Chip Electrophoresis	Region	HB	HC	6	**0.000**
Tuna 2013	Turkey	Caucasian	87/75	PCR-RFLP	Age, Region	HB	HC	5.5	0.986

HC, Histologically confirmed; PC, Pathologically confirmed; NA, Not available; PB, Population–based; HB, Hospital–based; HWE, Hardy–Weinberg equilibrium in control population; PCR–RFLP, Polymerase chain reaction-restriction fragment length polymorphism; PCR-SSCP, Polymerase chain reaction–single strand conformation polymorphism; ARMS-PCR, Amplification Refractory Mutation System-Polymerase Chain Reaction; MALDI-TOF, Matrix-assisted laser desorption/ionization time-of-flight; FISH, Fluorescence in situ hybridization

### Meta-analysis

**Figure 2:**
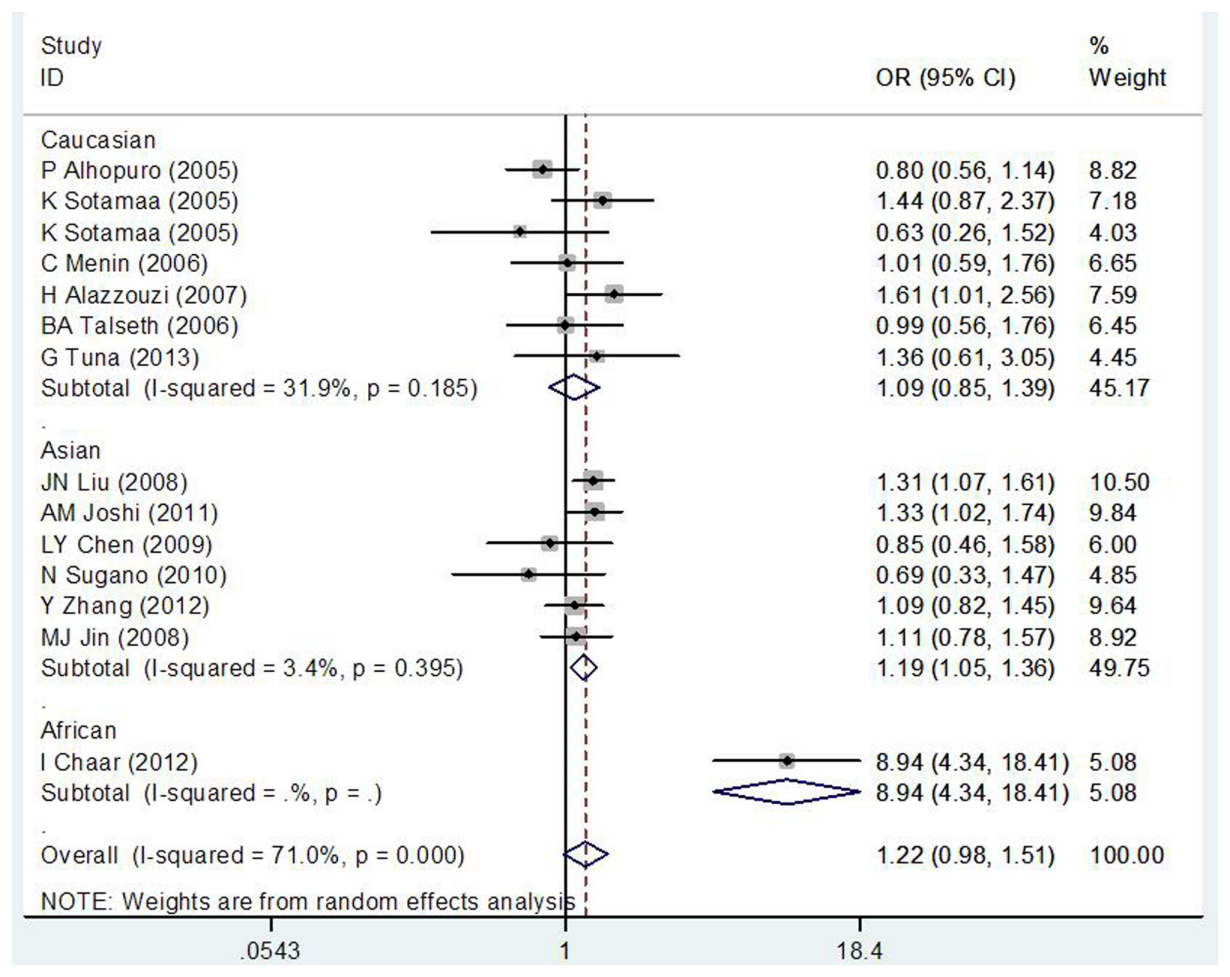
Subgroup analysis by ethnicity in the meta-analysis on the association between MDM2 SNP309 polymorphism and CRC risk using a random-effect model (additive model TG versus TT).

As shown in Table 3, we did not find a significant association between MDM2 SNP309 polymorphism and CRC risk in overall populations (GG vs. TT: OR = 1.086, 95% CI = 0.773–1.525, P = 0.634; GT vs. TT: OR = 1.217, 95% CI = 0.979–1.512, P= 0.077; GG+ GT vs. TT: OR = 1.176, 95% CI = 0.936–1.478, P= 0.163; GG vs. GT+ TT: OR = 0.959, 95% CI = 0.748–1.230, P=0.743). However, in subgroup analysis by ethnicity, the results of our study suggested that there was a positive correlation between the MDM2 SNP309 polymorphism and CRC risk in Asian population (TG vs. TT: OR = 1.197, 95% CI = 1.055–1.358, P=0.005; GG+TG vs. TT: OR = 1.246, 95% CI = 1.106–1.404, P=0.000) and African population (GG vs. TT: OR = 8.665, 95% CI = 4.139–18.141, P = 0.000; GT vs. TT: OR = 8.935, 95% CI = 4.337–18.409, P= 0.000; GG+ GT vs. TT: OR = 8.812, 95% CI = 4.436–17.506, P= 0.000; GG vs. GT+ TT: OR = 1.843, 95% CI = 1.167–2.908, P=0.009; Figure 2). Moreover, in the stratified analysis by HWE in controls, our result indicated a significant association between the MDM2 SNP309 polymorphism and CRC incidence in the studies consistent with HWE (TG vs. TT: OR = 1.166, 95% CI = 1.037–1.311, P= 0.010). However, in subgroup analysis by p53 mutation status and gender, we did not detect any significant association between this polymorphism and the risk of CRC in all genetic models.

**Table 3:** Meta-analysis of MDM2 SNP309 polymorphism and CRC risk.

Analysis	No. of studies	Homozygote (GG vs. TT)			Heterozygote (TG vs. TT)				Dominant model (GG+TG vs. TT)				Recessive model (GG vs. TG+TT)	
		OR (95% CI)	*P/P*_h_		OR (95% CI)		*P/P*_h_		OR (95% CI)		*P/P*_h_		OR (95% CI)	*P/P*_h_
Overall	14	1.086 (0.773-1.525)	0.634/0.000		1.217 (0.979-1.512)	0.077/0.000			1.176 (0.936-1.478)	0.163/0.000			0.959 (0.748-1.230)	0.743/0.000	
Ethnicity															
Caucasian	7	0.848 (0.643-1.118)	0.242/0.307		1.071 (0.881-1.301)	0.494/0.185			1.016 (0.844-1.223)	0.865/0.200			0.812 (0.635-1.038)	0.097/0.136	
Asian	6	1.086 (0.729-1.618)	0.684/0.000		**1.197 (1.055-1.358)**	**0**.**005**/0.395			**1.246 (1.106-1.404)**	**0**.**000**/0.046			1.027 (0.729-1.447)	0.880/0.000	
African	1	**8.665 (4.139-18.141)**	**0**.000/—		**8.935 (4.337-18.409)**	**0**.000/—			**8.812 (4.436-17.506)**	**0.000/**—			**1.843 (1.167-2.908)**	**0**.009/—	
p53 mutation status															
Positive	2	0.777 (0.426-1.418)	0.411/0.138		1.209 (0.824-1.773)	0.332/0.344			1.100 (0.768-1.575)	0.604/0.225			0.709 (0.398-1.263)	0.243/0.196	
Negative	2	0.884 (0.482-1.620)	0.690/0.668		1.409 (0.956-2.075)	0.083/0.340			1.274 (0.884-1.835)	0.194/0.515			0.762 (0.429-1.352)	0.353/0.457	
Gender															
Female	3	1.030 (0.736-1.442)	0.862/0.206		1.003 (0.760-1.325)	0.981/0.813			1.011 (0.776-1.317)	0.937/0.936			0.898 (0.517-1.560)	0.702/0.058	
Male	3	0.978 (0.727-1.317)	0.884/0.219		1.110 (0.603-2.042)	0.737/0.008			1.026 (0.601-1.753)	0.925/0.017			0.852 (0.672-1.080)	0.186/0.437	
HWE in controls															
Yes	9	1.054 (0.763-1.457)	0.751/0.000		**1.166 (1.037-1.311)**	**0**.**010**/0.261			1.124 (0.942-1.342)	0.195/0.046			0.968 (0.723-1.296)	0.829/0.000	
No	5	1.186 (0.422-3.335)	0.746/0.000		1.535 (0.749-3.145)	0.241/0.000			1.421 (0.675-2.992)	0.355/0.000			0.921 (0.529-1.604)	0.771/0.002	

*P*_h_ P values of Q-test for heterogeneity test. OR, odds ratio; CI, confidence intervals; HWE, Hardy–Weinberg equilibrium

### Test of heterogeneity

**Figure 3:**
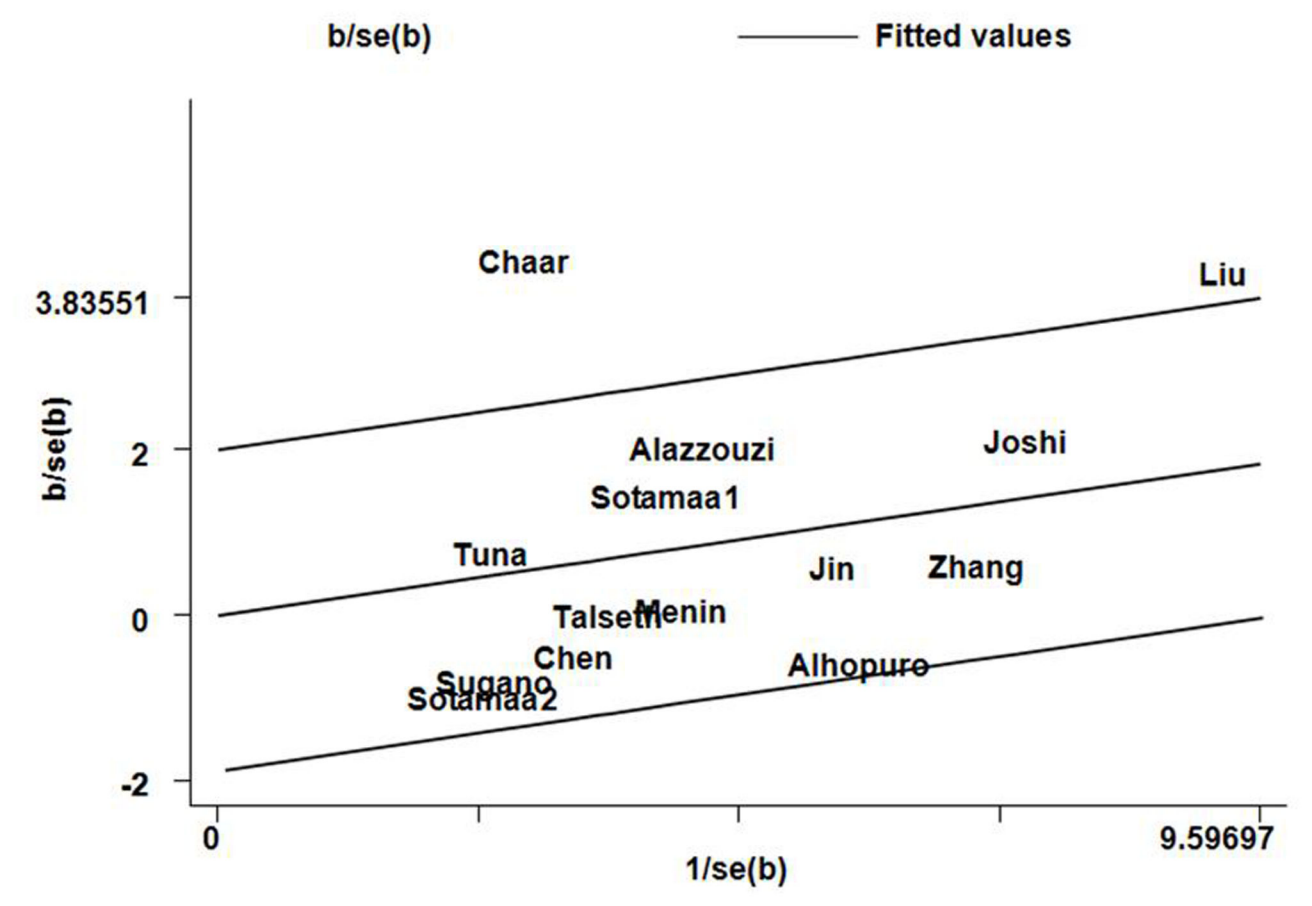
Galbraith plots of MDM2 SNP309 polymorphism and CRC risk in additive model TG versus TT. The studies of Chaar et al. and Liu et al. were spotted as outliers.

Significant heterogeneity was observed in the association analysis between the MDM2 SNP309 polymorphism and CRC risk in the overall populations in all comparisons (GG vs. TT: *P*_*Q*_ = 0.000; GT vs. TT: *P*_*Q*_ = 0.000; GG+ GT vs. TT: *P*_*Q*_ = 0.000; GG vs. GT+ TT: *P*_*Q*_ = 0.000; Table 3). To explore the sources of heterogeneity, we performed metaregression and subgroup analyses. Metaregression analysis of data showed that the ethnicity was the major source which contributed to heterogeneity. The Genotyping methods, Source of control, Quality scores, and CRC diagnosis were not effect modifiers. Subsequently, we performed subgroup analyses stratified by ethnicity. However, heterogeneity still existed in most of the genetic comparison models among Asians (GG vs. TT: *P*_*Q*_ = 0.000; GG+ GT vs. TT: *P*_*Q*_ = 0.046; GG vs. GT+ TT: *P*_*Q*_ = 0.000; Table 3). To further investigate the heterogeneity, we performed Galbraith plots analysis to identify the outliers which might contribute to the heterogeneity. Our results showed that the studies Liu et al. [30] and Chaar et al. [34] were outliers in additive models GG vs. TT and GT vs. TT (Figure 3), recessive model GG vs. GT+ TT, and dominant model GG+ GT vs. TT in the overall populations. All *I* ^2^ values decreased obviously and *P*_*Q*_ values were greater than 0.10 after excluding the two studies Liu et al. [30] and Chaar et al. [34] in all genetic comparison models in the overall populations (GG vs. TT: *P*_*Q*_ = 0.172; GT vs. TT: *P*_*Q*_ = 0.297; GG+ GT vs. TT: *P*_*Q*_ = 0.280; GG vs. GT+ TT: *P*_*Q*_ = 0.185), Asians (GG vs. TT: *P*_*Q*_ = 0.132; GG+ GT vs. TT: *P*_*Q*_ = 0.371; GG vs. GT+ TT: *P*_*Q*_ = 0.119), and studies consistent with HWE (GG vs. TT: *P*_*Q*_ = 0.347; GG+ GT vs. TT: *P*_*Q*_ = 0.412; GG vs. GT+ TT: *P*_*Q*_ = 0.202). The signiﬁcance of the summary ORs for MDM2 SNP309 polymorphism in different comparison models in the overall population and subgroup analyses were not inﬂuenced by omitting the two studies.

### Sensitivity analysis

**Figure 4:**
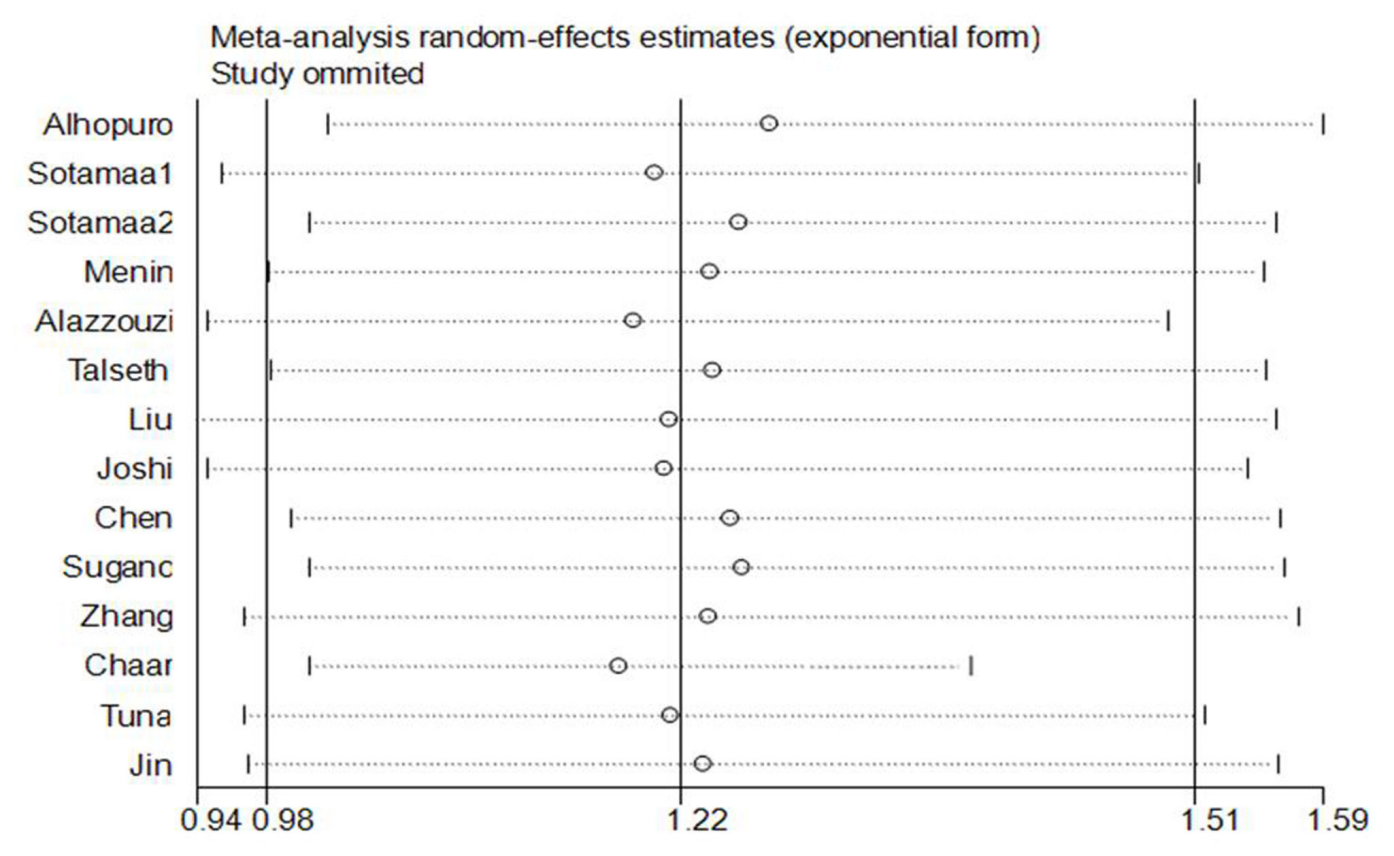
Inﬂuence analysis for additive model TG versus TT in the overall meta-analysis. This ﬁgure shows the inﬂuence of individual studies on the summary OR. The middle vertical axis indicates the overall OR and the two vertical axes indicate its 95% CI. Every hollow round indicates the pooled OR when the left study is omitted in this meta-analysis. The two ends of every broken line represent the 95% CI.

Sensitivity analysis was performed to assess the influence of each individual study on the pooled OR by sequential removal of individual studies. The results suggested that no individual study significantly affected the pooled ORs (Figure 4). Sensitivity analysis by excluding HWE-violating studies did not perturb the overall results.

### Publication bias

**Figure 5:**
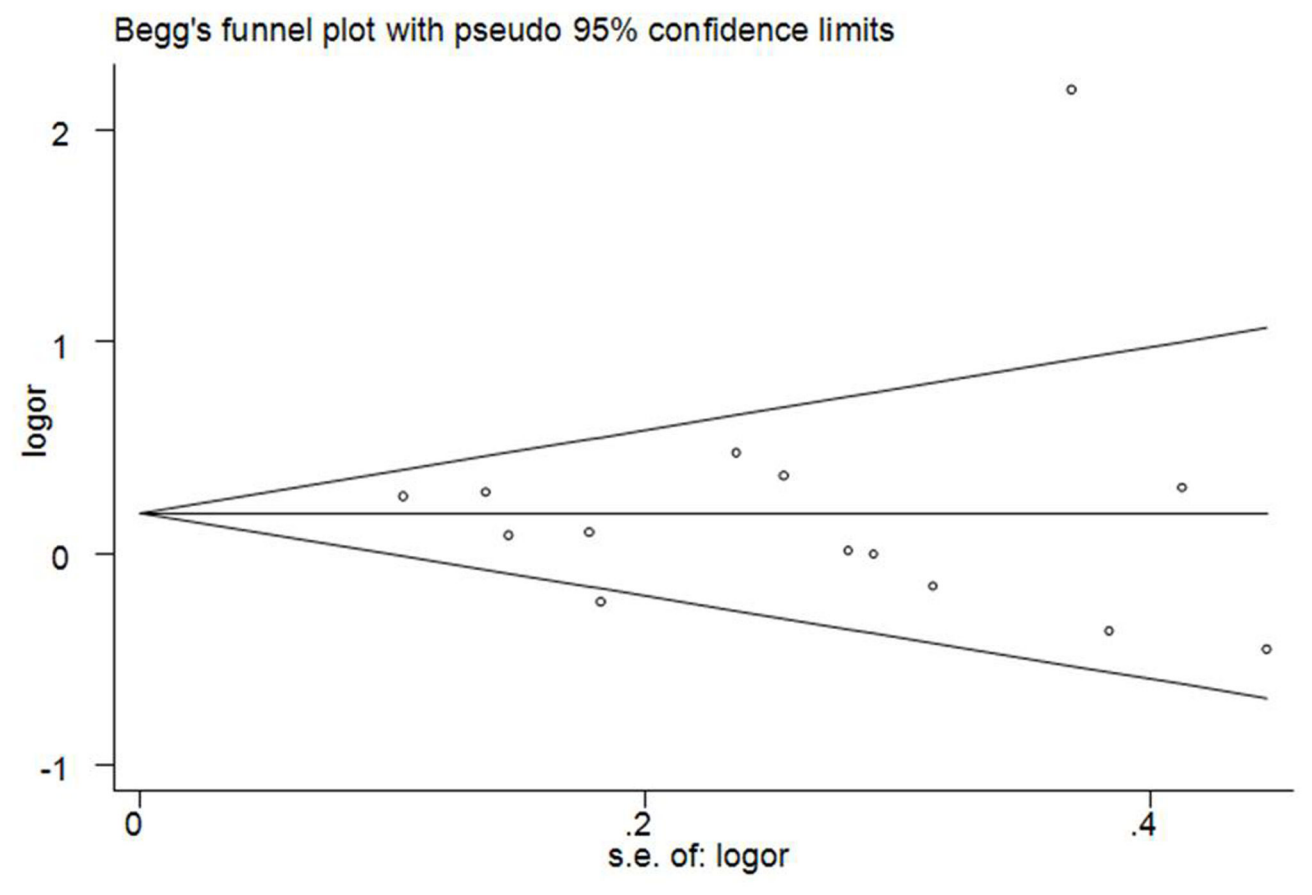
Funnel plots for publication bias of the meta-analysis on the association between MDM2 SNP309 polymorphism and CRC risk of the overall populations (additive model TG versus TT).

Begg’s funnel plot and Egger’s test were performed to access the publication bias of literatures in this meta-analysis. The shapes of Funnel plot did not reveal obvious evidence of asymmetry, and all the p values of Egger’s tests were more than 0.05, providing statistical evidence of the funnel plots’ symmetry (Figure 5). Thus, the results above suggested that publication bias was not evident in this meta-analysis.

## Discussion

Previous studies investigating the association between the MDM2 SNP309 polymorphism with CRC risk have provided inconsistent results, and most of those studies involved no more than a few hundred CRC cases, which is too few to assess any genetic effects reliably. Meta-analysis has been recognized as an important tool to more precisely define the effect of selected genetic polymorphisms on the risk for disease and to identify potentially important sources of between-study heterogeneity. A meta-analysis of 8 studies by Cao et al. [12] in 2011 showed that the MDM2 SNP309 polymorphism might be a risk factor for CRC, the variant genotype was associated with a significant increased CRC risk among the overall populations (GT vs. TT: OR=1.19, 95% CI=1.06–1.35) and Asians (GT vs. TT: OR=1.28, 95% CI=1.10–1.50). Another meta-analysis including 7 studies by Fang et al. [11], performed almost at the same time and quite similar in methods, drew opposite conclusion. The authors revealed that the MDM2 SNP309 polymorphism played a protective role in CRC susceptibility in Asians (GG vs. TT: OR = 0.51, 95% CI = 0.41–0.64; GG vs. TG: OR = 0.64, 95% CI = 0.53–0.78; GG+TG vs. TT: OR = 0.59, 95% CI = 0.49–0.71; GG vs. TG+TT: OR = 0.69, 95% CI = 0.57–0.82). The previous meta-analyses did not cover all eligible studies especially studies published in Chinese. Some studies were only indexed in the CBM database but not indexed in the databases selected in the meta-analyses by Cao et al. and Fang et al., which could lead to location bias and might bias the effect estimate of a meta-analysis. Furthermore, a number of new case–control studies have been published after these two meta-analyses. Hence, to provide the most comprehensive assessment of the associations between the MDM2 SNP309 polymorphism with CRC risk, we performed an updated meta-analysis of all available studies. The meta-analysis was carried out by critically reviewing 14 individual case–control studies on MDM2 SNP309 polymorphism and CRC risk. Subgroup analyses were mainly done by ethnicity, p53 mutation status, gender, and HWE in controls. Heterogeneity analysis and sensitivity analysis were also critically performed to ensure the epidemiological credibility of this meta-analysis. We found that the MDM2 SNP309 polymorphism was associated with an increased CRC risk among Asians (TG vs. TT: OR = 1.197, 95% CI = 1.055–1.358, P=0.005; GG+TG vs. TT: OR = 1.246, 95% CI = 1.106–1.404, P=0.000), which was in accordance with the previously published meta-analysis by Cao et al. [12].

P53 is the most frequently mutated gene in human tumors [37]. In view of the robust effect of p53 mutation in carcinogenesis, the impact of MDM2 SNP309 polymorphism on the Li-Fraumeni syndrome has been characterized in several studies [38,39]. Furthermore, significant higher risk associated with GG genotype of MDM2 SNP309 polymorphism among the p53 mutation-positive subgroup have been found in lung cancer [40] and gastric cancer [41], showing that SNP309 G allele could accelerate tumor formation and cause the occurrence of multiple primary tumors in a lifetime for P53 mutation carriers [9,38]. Therefore, it is necessary to incorporate the mutation status of p53 when explore the effects of MDM2 SNP309 on tumors. Thus far, there were only two studies on the association between MDM2 SNP309 polymorphism and CRC risk according to p53 mutation status in cases available for pooled analysis [27,28]. However, no significant discrepancy was found in the two p53 mutation status subgroups, probably because of the insufficient statistical power. Further functional and molecular epidemiologic studies were suggested to explore the joint/interaction effects between functional polymorphisms in p53-MDM2–related genes and p53 mutation status in CRC susceptibility.

When stratified by ethnicity, the MDM2 SNP309 polymorphism presented a risk factor for CRC in Asian and African populations, but not in Europeans. Actually, it might not be uncommon for the same polymorphism playing different roles in cancer susceptibility among different ethnic populations. In Asians and Africans, the differences in genetic backgrounds and the environment they lived in may inﬂuence the association between the MDM2 SNP309 polymorphism and CRC risk. In addition, owing to the limited number of relevant studies among African population included in this meta-analysis, the observed positive association between MDM2 SNP309 polymorphism and CRC risk in Africans is likely to be caused by chance because study with small sample sizes may have insufficient statistical power to detect a slight effect or may have generated a fluctuated risk estimate. Currently there is only one study [34] on MDM2 SNP309 polymorphism and CRC risk among African population, and the genotype distributions in the control population of this study was deviated from HWE. Therefore, the positive results of the African population should be interpreted with caution.

It seemed that selection bias could have played a role because the genotype distribution of the MDM2 SNP309 polymorphism among control subjects disobeyed the law of HWE in five studies [26,28,32,34,36]. It is widely believed that deviation from HWE may be as a result of genetic reasons including non-random mating, or the alleles reflect recent mutations that have not reached equilibrium, as well as methodological reasons including biased selection of subjects from the population or genotyping errors [42,43]. Because of the reasons of disequilibrium, the results of genetic association studies might be spurious if the distribution of genotypes in the control groups were not in HWE [44,45]. Hence, we carried out subgroup analysis by HWE in controls. When excluding the studies that were not in HWE, the results were persistent and robust, suggesting that this factor probably had little effect on the overall estimates.

Evidence suggests that estrogen receptors have been widely detected in cancer cells, indicating that sex steroid may play a critical role in the pathogenesis of cancers [46,47]. Besides, MDM2 may act as a strong contributor via the P53-independent pathway during the process of estrogen-induced cell proliferation [48]. MDM2 can induce expression of the p65 subunit of NF-kB, which is an anti-apoptotic factor expressed in neoplastic cells [49]. In addition, SNP309 of MDM2 increases the binding affinity for Sp1, a coactivator of receptors for multiple hormones including estrogen. It could potentially affect the hormone-dependent regulation of MDM2 transcription and result in further elevation of the MDM2 protein levels [50,51]. Thus, the MDM2 SNP309 polymorphism might accelerate carcinogenesis of colorectal tissues in a gender-specific manner [52]. Therefore, we carried out subgroup analysis according to gender. However, no significant associations were found in both Female and Male subgroups for all genetic models in our meta-analysis. The results should be interpreted with care because of the limited numbers of the original studies. Therefore, further studies concerning stratification for gender are needed to increase power for the association estimation.

Heterogeneity is a potential problem when interpreting the results of a meta-analysis, and finding the sources of heterogeneity is one of the most important goals of meta-analysis [53]. In the present meta-analysis, significant between-study heterogeneity in the pooled analyses of total eligible studies was observed (all *P*_*Q*_ values were 0.000). To find the sources of heterogeneity, we performed metaregression and subgroup analyses. Metaregression analysis of data showed that the ethnicity was the major source which contributed to heterogeneity. The Genotyping methods, Source of control, Quality scores, and CRC diagnosis were not effect modifiers. Subgroup analysis by ethnicity showed that the heterogeneity was still significant in Asians. To further investigate the heterogeneity, Galbraith plots analysis was performed to identify the outliers which might contribute most to the heterogeneity. Our results showed that the studies of Liu et al. [30] and Chaar et al. [34] were outliers of all genetic comparison models in the overall populations. All *I* ^2^ values decreased lower than 50% and *P*_*Q*_ values were larger than 0.10 after excluding the studies of Liu et al. [30] and Chaar et al. [34] in all genetic comparison models in the overall populations and Asians. In addition, the summary ORs for the MDM2 SNP309 polymorphism in different comparison models in the overall population and subgroup analyses were not material change by omitting the two studies, indicating that our results were robust and reliable. The results indicated that the two studies might be the major source of the heterogeneity in the meta-analysis.

Some possible limitations in this meta-analysis should be acknowledged. Firstly, in subgroup analysis by ethnicity, p53 mutation status, and gender, the sample size of population was relatively small for stratified analyses, which may lead to relatively weak power to detect the real relationship. Secondly, our results were based on unadjusted estimates. We did not perform the analysis adjusted for other covariates such as age, drinking status, cancer type, environment factors, and so on, because of the unavailable original data of the eligible studies.

In conclusion, our meta-analysis provided a more precise estimation based on larger sample size compared with the previous meta-analyses. Our study suggested that the MDM2 SNP309 polymorphism might contribute to CRC risk, especially in Asian populations. In order to further verify our findings, large well-designed epidemiological studies are warranted.

## Supporting Information

Checklist S1 **PRISMA checklist.**
(DOC)

Figure S1 **Flow diagram of included studies for this meta-analysis.**
(TIF)
