# Supplementary figures and images for: An Updated Meta-Analysis on the Association of MDM2 SNP309 Polymorphism with Colorectal Cancer Risk
Source: PLoS One. 2013 Sep 30;8(9):e76031. doi: 10.1371/journal.pone.0076031 (PMC3786895; doi:10.1371/journal.pone.0076031)

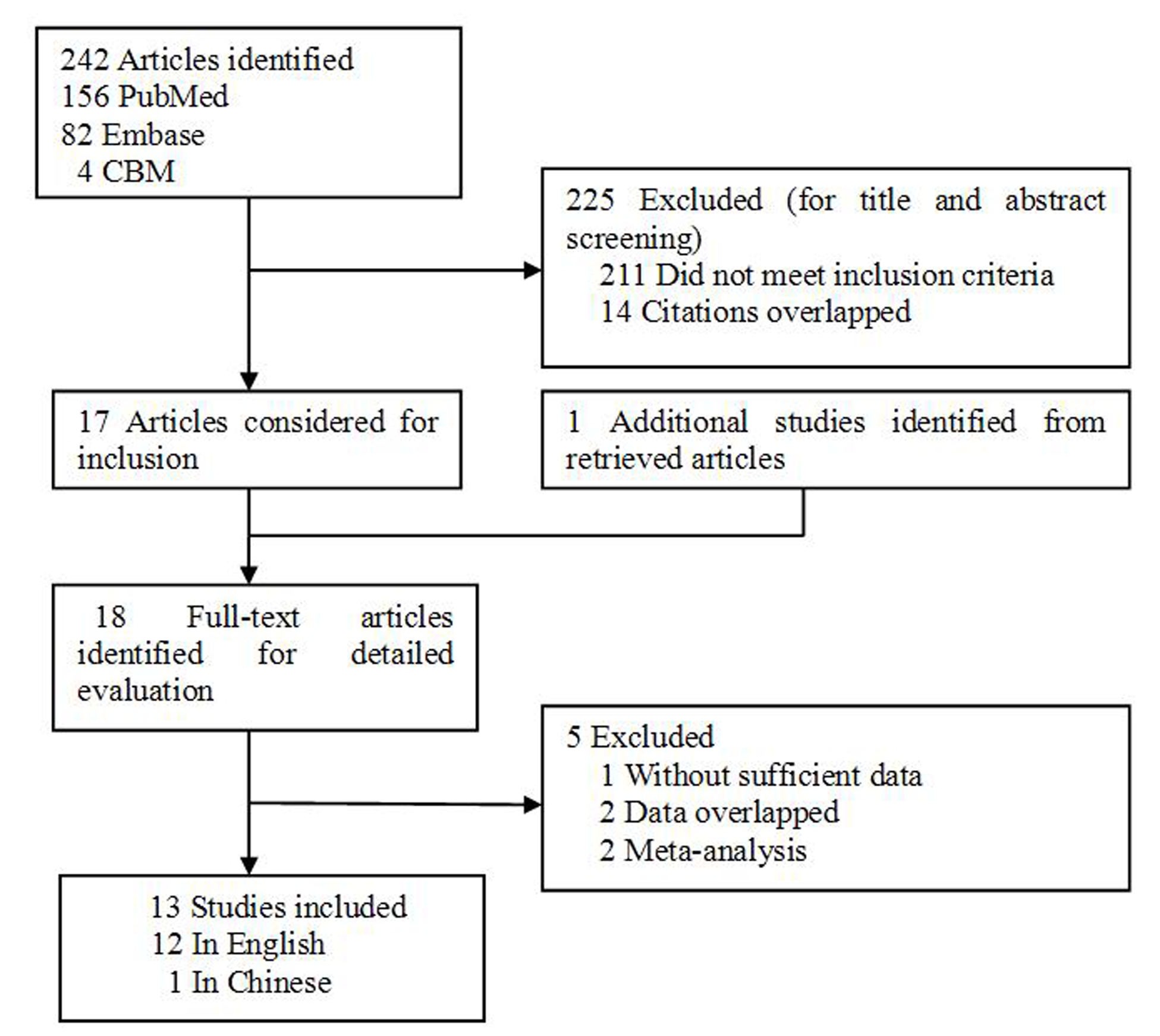

Supplement: Figure S1 — Flow diagram of included studies for this meta-analysis. (TIF) [file pone.0076031.s002.tif]
